# Association of Frailty Status with Staging and Mortality Risk of Cardiovascular-Kidney-Metabolic Syndrome in Middle-Aged and Older Populations: Insights from the 1999–2018 National Health and Nutrition Examination Survey

**DOI:** 10.3390/jcm14176008

**Published:** 2025-08-25

**Authors:** Zhenkun Yang, Shuang Wu, Yuanjie Li, Hongyu Liu, Manlin Zhao, Yang Xu, Yunyu Chen, Yang Chen, Gregory Y. H. Lip

**Affiliations:** 1 Department of Nephrology, China-Japan Friendship Hospital, Beijing 100029, China; yzk1584772@163.com; 2 National Center for Cardiovascular Disease, Fuwai Hospital, Chinese Academy of Medical Sciences, Peking Union Medical College, Beijing 100037, China; wushuang@fuwaihospital.org; 3 Department of Emergency Center, Fuwai Hospital, Chinese Academy of Medical Sciences, Peking Union Medical College, Beijing 100037, China; 4 Tianjin Research Institute of Anesthesiology, Department of Anesthesiology, Tianjin Medical University General Hospital, Tianjin 300052, China; liyuanjie0222@163.com; 5 Liverpool Centre for Cardiovascular Science at University of Liverpool, Liverpool John Moores University, Liverpool Heart and Chest Hospital, Liverpool L14 3PE, UK; hongyu.liu@liverpool.ac.uk (H.L.); manlin.zhao@liverpool.ac.uk (M.Z.); 6 Department of Cardiovascular Medicine, The Second Affiliated Hospital, Jiangxi Medical College, Nanchang University, Nanchang 330006, China; 7 Department of Cardiology, Beijing Anzhen Hospital, Capital Medical University, Engineering Research Center of Medical Devices for Cardiovascular Diseases, Ministry of Education, National Clinical Research Center for Cardiovascular Diseases, Beijing 100029, China; 8 Department of Cardiology, Tianjin Medical University General Hospital, Tianjin 300052, China; yuong1101@163.com; 9 Department of Medical Research, Taichung Veterans General Hospital, No. 1650, Sec. 4, Taiwan Boulevard, Xitun District, Taichung 407219, Taiwan; r01847021@gmail.com; 10 Cardiovascular Center, Taichung Veterans General Hospital, Taichung 407219, Taiwan; 11 Heart Rhythm Center, Division of Cardiology, Department of Medicine, Taipei Veterans General Hospital, Taipei 112201, Taiwan; 12 Cardiovascular Research Center, College of Medicine, National Chung Hsing University, Taichung 402202, Taiwan; 13 Department of Cardiovascular and Metabolic Medicine, Institute of Life Course and Medical Sciences, University of Liverpool, Liverpool L69 7ZX, UK; 14 Danish Centre for Health Services Research, Department of Clinical Medicine, Aalborg University, DK-9220 Aalborg, Denmark; 15 Department of Cardiology, Lipidology and Internal Medicine, Medical University of Bialystok, ul. Żurawia 14, 15-540 Bialystok, Poland

**Keywords:** frailty, cardiovascular-kidney-metabolic syndrome, staging, mortality, NHANES

## Abstract

**Background:** Cardiovascular-kidney-metabolic syndrome (CKM) represents a multisystem condition involving obesity, diabetes, chronic kidney disease, and cardiovascular diseases. Frailty, as measured by the Frailty Index (FI), is linked to adverse outcomes, but its association with CKM severity and mortality remains unclear. This study aimed to evaluate the relationship between frailty status, CKM staging, and mortality risk. **Methods:** We analysed data from 19,407 adults aged ≥ 45 years from NHANES 1999–2018. Frailty status was assessed using a 49-item Frailty Index (FI) and categorised as robust (FI ≤ 0.08), pre-frail (0.08 < FI < 0.25), or frail (FI ≥ 0.25). CKM was staged from 1 to 4 based on established clinical criteria. Multinomial logistic regression assessed the association between frailty status and CKM staging. Cox proportional hazards models evaluated the associations between frailty status and all-cause, cardiovascular, and non-cardiovascular mortality among CKM patients. **Results:** A total of 19,407 participants (median [IQR] age: 63.00 [54.00–72.00] years, 50.77% male), with 19,089 CKM patients. Frail individuals exhibited significantly higher odds of being assigned to advanced CKM stages. Over a median follow-up of 8.4 years, 4794 participants died. Kaplan–Meier curves and restricted cubic spline analyses demonstrated a clear gradient in mortality risk across frailty categories. Compared with the robust group, pre-frail and frail individuals had significantly higher risks of all-cause (HR = 1.47 and 2.83, respectively), cardiovascular (HR = 1.71 and 3.78), and non-cardiovascular mortality (HR = 1.40 and 2.57). **Conclusions:** Frailty status demonstrated a significant association with CKM staging and mortality outcomes. Early identification of frailty may help guide risk stratification and inform tailored interventions for individuals with CKM.

## 1. Introduction

The American Heart Association (AHA) recently introduced the cardiovascular-kidney-metabolic syndrome (CKM) initiative defined as a health disorder caused by the pathophysiological interactions between obesity, diabetes, chronic kidney disease (CKD), and cardiovascular diseases (CVD) [including heart failure, atrial fibrillation, coronary artery disease, stroke, and peripheral artery disease] [1]. Globally, CVD is the leading cause of death and disability, with an estimated prevalence of approximately 500 million people [2]. According to the International Diabetes Federation, the global population of type 2 diabetes patients is approximately 537 million, and this number is expected to rise to 643 million by 2030 [3]. The prevalence of CKD is approximately 10% to 15% of the total adult population [4]. The emerging concept of CKM emphasises exactly the important interactions among these major chronic diseases and highlights the need for comprehensive risk assessment and management strategies.

CKM syndrome is essentially a multisystem degenerative disease characterised by progressive impairment of cardiac, renal, and metabolic functions [5]. CVD such as heart failure and coronary artery disease contribute to reduced exercise tolerance and physical capacity in affected patients [6]. Additionally, CKM patients often experience chronic systemic inflammation driven by insulin resistance, hyperglycemia, and lipid metabolism disorders, which promote oxidative stress and cause cellular dysfunction [7]. Such metabolic dysregulation may cause a decline in muscle mass and strength, ultimately leading to sarcopenia, weakness, and decreased physical performance [8], which may further exacerbate the overall functional decline of CKM patients.

Physical weakness is typically assessed through indicators such as unintentional weight loss, muscle weakness, fatigue, slowness of movement, and reduced physical activity [9]. The frailty index (FI), by contrast, offers a more comprehensive evaluation of frailty by incorporating physiological, psychological, and functional health factors [10]. Higher FI scores have been associated with an increased incidence of various diseases and adverse health outcomes [11,12]. Despite its broad applicability, the relationship between FI and CKM staging remains poorly understood. In addition, evidence from a large meta-analysis supports the role of FI as a strong predictor of mortality [13], and FI has also been associated with increased mortality risk in some individuals with specific conditions, such as diabetes patients [14]. Despite its clinical relevance, particularly in middle-aged and older adults [15], the associations between frailty and mortality outcomes have rarely been explored in the context of CKM.

This study aimed to (1) assess the cross-sectional relationship between the frailty status and CKM staging, and (2) examine the associations between the frailty status and different mortality risks in CKM patients.

## 2. Methods

### 2.1. Data Sources

The data for our study were obtained from the National Health and Nutrition Examination Survey (NHANES) 1999–2018. NHANES is a national survey conducted by the Centers for Disease Control and Prevention aimed at assessing the health and nutritional status of United States residents. The database contains a wide range of health-related data, including examination results, laboratory tests, dietary intake, and health behaviors, which has been approved by the Ethics Review Board of the National Center for Health Statistics in the United States. Informed consent was obtained from each participant in the NHANES. This study complies with the Strengthening the Reporting of Observational Studies in Epidemiology (STROBE) guidelines (Supplementary Table S1).

### 2.2. Study Design and Participant Selection

In NHANES, we included 55,081 adults aged ≥ 20 years from ten survey cycles (1999–2018). After excluding participants with missing CKM-related variables (N = 17,949), pregnant individuals (N = 539), those aged < 45 years (N = 10,036), participants with other missing baseline data (N = 7131), and those with incomplete follow-up information (N = 19), a total of 19,407 participants aged ≥ 45 years were included for cross-sectional analysis of the association between FI and CKM staging. For the mortality outcome analysis, CKM patients (N = 19,089) from the NHANES 1999-018 cycles were followed up for survival status. Both all-cause and cause-specific mortality data were available in NHANES (Supplementary Figure S1).

### 2.3. Extracted Covariates

Covariates included demographic characteristics (age, sex, race/ethnicity, systolic blood pressure, diastolic blood pressure, height, weight, waist circumference [WC]), socioeconomic status (education level, marital status, poverty income ratio), lifestyle factors (smoking, alcohol use, physical activity), and laboratory indicators (glucose, hemoglobin A1c, triglycerides, total cholesterol, high-density lipoprotein cholesterol, creatinine, and urine albumin). Race and ethnicity were self-reported by participants and included categories such as non-Hispanic White, non-Hispanic Black, Mexican American, Hispanic, and others. Body mass index (BMI) was determined by dividing weight (kg) by the square of height (m^2^). Smoking status was classified into three groups: never smokers (fewer than 100 cigarettes in their lifetime), former smokers (at least 100 cigarettes but not currently smoking), and current smokers (at least 100 cigarettes and still smoking).

### 2.4. Definitions of CKM

The criteria for defining CKM are detailed in Supplementary Table S2. Following the AHA classification framework and previous publications, CKM was staged from 1 to 4 based on clinical and subclinical indicators (see Supplementary Table S3) [1,16,17]. Subclinical cardiovascular disease risk was estimated using the AHA PREVENT equations to calculate 10-year CVD risk (Supplementary Table S4) [18]. Kidney function was categorised according to the guidelines of Kidney Disease: Improving Global Outcomes [19]. The urine albumin-to-creatinine ratio was determined by dividing urine albumin (µg/mL) by urine creatinine (mg/dL) and multiplying by 100 [20].

### 2.5. Assessment of Frailty Index

Frailty status was evaluated by the FI, which was calculated by the accumulation of multiple age-related health deficits. The FI was constructed using 49 available assessment parameters (Supplementary Table S5), in cases where some items were missing. All variables were equally weighted, following the deficit accumulation approach, where each deficit contributes one point (i.e., weight = 1) to the total score. The FI was calculated as the number of present deficits divided by the total number of considered deficits, resulting in a continuous score between 0 and 1, following previously published method [16].

### 2.6. Mortality Outcomes

The mortality outcomes included all-cause mortality, cardiovascular mortality, and non-cardiovascular mortality. The mortality data were obtained from the Centers for Disease Control and Prevention website, updated until 31 December 2019. Causes of death were identified using the 10th Revision of the International Statistical Classification of Diseases and Related Health Problems (ICD-10). Follow-up time is from the interview to the date of the last follow-up or death.

### 2.7. Statistical Analysis

There were no missing values after excluding participants with missing covariate data. For continuous variables, the mean ± standard deviation or the median and interquartile range (IQR) were reported, depending on the data distribution. Group differences were analysed using one-way analysis of variance or Kruskal-Wallis tests, as appropriate. Differences in categorical variables among groups were assessed using Fisher’s exact or Chi-squared test, and results were expressed as counts and percentages.

The frailty status was categorized as follows: robust (FI ≤ 0.08), pre-frail (0.08 < FI < 0.25), and frail (FI ≥ 0.25). The cut-off values used to categorise frailty status were adopted from previous large-scale studies where these thresholds demonstrated strong predictive validity for adverse outcomes such as hospitalization, disability, and mortality [21,22]. These thresholds have been widely used and provide a meaningful clinical stratification of frailty risk. CKM staging was initially treated as an ordinal variable to explore its association with FI status. However, due to a violation of the parallel lines assumption, multinomial logistic regression was used instead. The robust group served as the reference, and odds ratio (OR) with 95% confidence interval (CI) were reported. The analysis adjusted for potential confounders, including age, sex, race and ethnicity, BMI, WC, marital status, education level, smoking status, alcohol consumption, physical activity, and poverty income ratio. The associations between frailty status and mortality outcomes were then assessed using Cox proportional hazards models, with prior verification of the proportional hazards assumption and adjustment for the previously described confounders. Hazard ratio (HR) with 95% CI were reported using the robust group as the reference. Furthermore, Kaplan-Meier survival curves were constructed to display cumulative mortality events across frailty status groups in CKM patients, with statistical differences evaluated using the log-rank test. Moreover, RCS analyses were utilised to investigate the potential nonlinear associations between continuous FI and mortality outcomes among CKM patients. Subgroup analyses and interactions were additionally conducted to examine the impact of age (<65 vs. ≥65 years), sex, BMI (<30 vs. ≥30 kg/m^2^) on the associations between frailty status and mortality outcomes in CKM patients.

Several sensitivity analyses were conducted in both database: (i) Because the FI cut-off values for defining frailty status were controversial, we used another two common cut-off values, including: (1) frail, pre-frail, and robust were defined as FI > 0.21, 0.10 < FI ≤ 0.21, and FI ≤ 0.10; (2) frail, pre-frail, and robust were defined as FI ≥ 0.25, 0.10 < FI < 0.25, and FI ≤ 0.10 [21,22], to define frailty status to conduct sensitivity analyses using Cox models for three different mortality outcomes; (ii) To reduce the impact of severe comorbidities on mortality, the Cox analyses were repeated after excluding participants with cancer; (iii) to minimize the reverse causality, the Cox analyses were repeated after excluding participants who died in the first two-year follow-up.

All statistical analyses were performed using SPSS Statistics (version 27, IBM Corp., Armonk, NY, USA) and R software (version 4.4.2, R Foundation for Statistical Computing, Vienna, Austria). A two-tailed *p*-value < 0.05 was considered statistically significant.

## 3. Results

### 3.1. Baseline Characteristics

Finally, 19,407 eligible participants were included in this analysis (median [IQR] age: 63.00 [54.00–72.00] years, 50.77% male, median [IQR] follow-up: 8.41 [4.50–12.67] years). This cohort comprised 19,089 CKM patients and 318 non-CKM individuals, and 4794 participants died during follow-up. Table 1 presents baseline characteristics of CKM patients stratified by frailty status, and Supplementary Table S6 presents baseline characteristics of whole cohort by CKM stages. Participants classified as frail were generally older, with higher BMI and larger WC, while also exhibiting lower proportions of smoking and alcohol consumption, and reduced engagement in vigorous physical activity (all *p* < 0.05).

### 3.2. Relationship Between Frailty Status and CKM-Staging Progression

**Figure 1:**
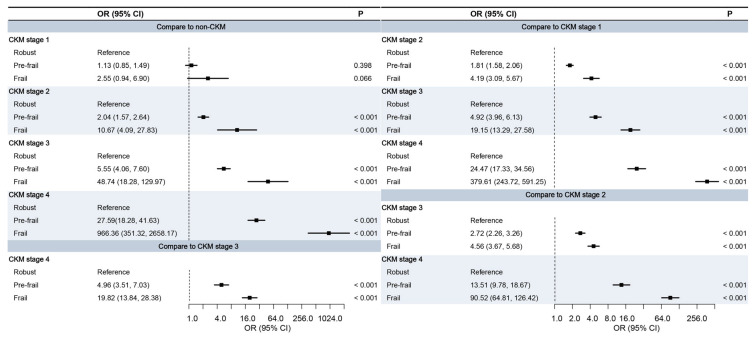
Association between frailty index and CKM staging (stages 0–4). Robust (FI ≤ 0.08), Pre-frail (0.08 < FI < 0.25), and Frail (FI ≥ 0.25). *p* values from multinomial Logistic regression models. CI, confidence interval; CKM, cardiovascular-kidney-metabolic syndrome; OR, odds ratio.

Compared to robust individuals, frail participants had markedly higher odds of being classified into more advanced CKM stages (Figure 1). For example, frail individuals had over 10 times the odds of being in CKM stage 2 (OR = 10.67, 95% CI: 4.09–27.83) and nearly 49 times the odds for stage 3 (OR = 48.74, 95% CI: 18.28–129.97) compared to non-CKM participants. In CKM stage 4, the odds increased dramatically (OR = 966.36, 95% CI: 351.32–2658.17). The extremely high ORs observed in the frail group, particularly at advanced CKM stages, may partly reflect quasi-complete separation due to the strong clustering of frail individuals in later CKM stages. As shown in Table 1, nearly one-third of the frail participants were classified as CKM stage 4, compared with only 0.5% in the robust group.

### 3.3. Relationship Between Frailty Status and Mortality Outcomes in CKM Patients

**Figure 2:**
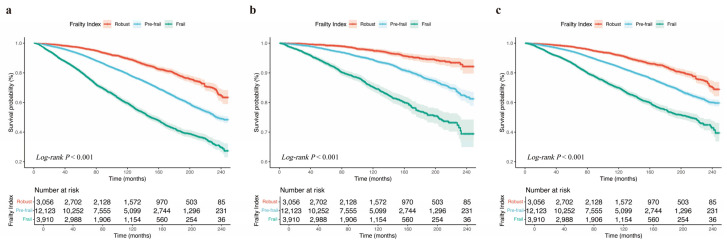
Kaplan-Meier survival curves for mortality outcomes across frailty index in CKM patients. (**a**) All-cause mortality, (**b**) cardiovascular mortality, (**c**) non-cardiovascular mortality. Robust (FI ≤ 0.08), Pre-frail (0.08 < FI < 0.25), and Frail (FI ≥ 0.25). Log-rank *p* values from log-rank test. CKM, cardiovascular-kidney-metabolic syndrome.

**Figure 3:**
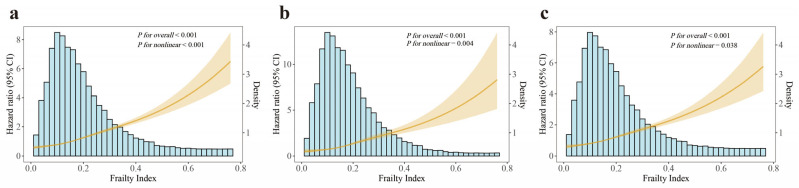
Restricted cubic spline analyses for associations between frailty index and mortality outcomes in CKM patients. (**a**) All-cause mortality, (**b**) cardiovascular mortality, (**c**) non-cardiovascular mortality. *p* values from multivariable Cox proportional hazards models. CI, confidence interval; CKM, cardiovascular-kidney-metabolic syndrome; HR, hazard ratio.

Supplementary Figure S2 illustrated a progressive increase in all-cause, cardiovascular, and non-cardiovascular mortality across frailty status groups (all *P* < 0.001). The Kaplan-Meier curves showed that for mortality outcomes the survival probability of CKM patients across different frail status groups (Figure 2; all *Log-rank P* < 0.001). Additionally, RCS analyses showed that FI was a significant nonlinear relationship with mortality outcomes (Figure 3; all-cause mortality: *P-for-nonlinear* < 0.001; cardiovascular mortality: *P-for-nonlinear* = 0.004; and non-cardiovascular mortality: *P-for-nonlinear* = 0.038).

From Table 2, after adjusting for confounders, multivariate Cox proportional hazards models analyses indicated that compared to robust group, pre-frail and frail groups were associated with higher risk of all-cause mortality (pre-frail: HR = 1.47, 95% CI: 1.32–1.62; frail: HR = 2.83, 95% CI: 2.53–3.17), cardiovascular mortality (pre-frail: HR = 1.71, 95% CI: 1.37–2.12; frail: HR = 3.78, 95% CI: 3.00–4.76), and non-cardiovascular mortality (pre-frail: HR = 1.40, 95% CI: 1.25–1.57; frail: HR = 2.57, 95% CI: 2.26–2.91).

### 3.4. Subgroup Analysis

In the stratified analyses, similar results were observed irrespective of subgroup classifications by age, sex, BMI, or CKM stage (Supplementary Figure S3). The risks of all-cause, cardiovascular, and non-cardiovascular mortality were significantly higher in the pre-frail and frail groups compared to the robust group. Although significant interactions in the age subgroups were observed between frailty status and mortality outcomes (all *p-interaction* < 0.05), the effect direction of the associations remained consistent.

### 3.5. Sensitivity Analysis

In the sensitivity analysis, the associations of frailty status changes with mortality outcomes were also consistent after further adjusting for using another two FI cut-off values to define frailty status (Supplementary Table S7). After excluding patients with a history of cancer (2876 patients) and CKM patients who died within two years of follow-up (548 patients), the associations of frailty status changes with mortality outcomes were also consistent (Supplementary Table S7).

## 4. Discussion

In this nationally representative cohort of U.S. adults, we found that frailty status was strongly associated with both the severity of CKM and increased risk of all-cause, cardiovascular, and non-cardiovascular mortality in CKM patients. Individuals classified as frail were significantly more likely to be in advanced CKM stages, and frailty status was a strong, independent predictor of long-term mortality in CKM patients. These associations remained robust across multiple sensitivity and subgroup analyses. Our findings underscore the clinical relevance of frailty as a stratification tool for identifying high-risk individuals within the CKM population, even at the early stages.

The significant potential association between the frailty status and adverse outcomes may result from the combined effect of multiple pathophysiological mechanisms. CKM patients are often in a state of chronic systemic inflammation, where oxidative stress induced by insulin resistance, hyperglycemia, and lipid metabolism disorders leads to cellular dysfunction, further exacerbating degenerative changes in the cardiac, renal, and metabolic systems [23,24]. CKM patients classified as pre-frail or frail often experience more severe inflammation and oxidative stress, accelerating disease progression and leading to higher mortality risks. Additionally, advanced CKM stage patients often exhibit significant cardiorenal and metabolic dysfunction, which amplifies the negative impact of the frailty on disease prognosis [24]. The frailty status reflects the degree of multisystem functional decline, including immune system deterioration, loss of muscle mass and strength, and abnormalities in metabolic regulation [9]. In this context, individuals with higher skeletal muscle mass and strength tend to have a better quality of life and lower mortality rates [25]. On the other hand, sarcopenia increases the rates of infection, hospitalization duration, mobility limitations, and mortality [26]. Particularly in the elderly, malnutrition and immune dysfunction can increase the risk of infections, pressure ulcers, morbidity, and mortality [27].

Unlike previous studies, this study comprehensively analyzed for the first time the relationship between the frailty status and CKM stage progression as well as its impact on different types of mortality risks. Previous studies primarily focused on the impact of the frailty status on specific individual diseases, such as diabetes [14], depression [28], and chronic obstructive pulmonary disease [29]. In contrast, our study extended the application of the frailty status to a multisystem condition, demonstrating its strong association with the severity classification of CKM. Furthermore, our findings confirmed the robust relationship between frailty status and mortality risk. A meta-analysis by Gotaro et al. demonstrated that higher FI groups were significantly associated with higher mortality risk (pooled HR for every 0.01 increase in the FI = 1.039, 95% CI: 1.033–1.044, *p* < 0.001; pooled OR for every 0.01 increase in the FI = 1.054, 95% CI: 1.040–1.068, *p* < 0.001) [13]. Xu et al. identified a potential bidirectional causal link between higher FI levels and CVD risk, highlighting shared etiological pathways [30]. A study from China indicated that for each 0.1 increment of the FI, the corresponding HRs for the risk of death were 1.89 (95% CI: 1.83–1.94) for ischemic heart disease, 1.84 (95% CI: 1.79–1.89) for cerebrovascular disease, and 1.78 (95% CI: 1.73–1.83) for all other causes [31]. Importantly, our study, leveraging a larger sample size and extensive subgroup analyses, further clarified the specific role of frailty status across CKM stages, providing stronger evidence to support its clinical utility.

The results of this study have significant clinical implications, frailty status can help clinicians identify high-risk individuals among CKM patients at an earlier stage. Prefrailty (the early stage of frailty) already poses a higher risk, and once frailty is identified in a patient, the risk of cardiovascular mortality increases sharply [32]. Furthermore, frailty status was shown to be a valuable indicator of disease severity and prognosis in CKM patients, supporting its potential role in guiding personalised management strategies and mitigating disease progression. In particular, assessing the degree of frailty in elderly patients with cardiovascular disease is crucial to evaluate the risks of functional decline, loss of independence, and mortality [33]. Our findings also provide a new direction for future CKM syndrome intervention research, focusing on whether improving frailty status could slow CKM progression and reduce mortality risk of CKM. Integrating frailty screening into CKM care pathways may enhance early identification of vulnerable patients and guide individualized interventions. The use of a simple, routinely collected frailty index could be embedded into electronic health records and reviewed during nephrology or multidisciplinary clinics. Interventions such as resistance and balance training, tailored nutritional supplementation, and comprehensive multimorbidity management can improve physical function and reduce adverse outcomes in frail individuals [34]. Future interventional studies are needed to evaluate the effectiveness of such strategies in CKM populations. Further support for the clinical relevance of frailty in older individuals with CKM can be drawn from the Miguel Camafort’s work [35]. This work emphasizes that hypertension and hypertensive heart disease frequently co-occur with aging, comorbidities, and functional decline, and underscores the necessity of individualized blood pressure management strategies in older, vulnerable populations. Integrating frailty assessment into CKM management could facilitate more nuanced cardiovascular risk stratification and guide therapeutic interventions such as tailored blood pressure control, optimally balancing risks and benefits.

### Limitation

This study has several limitations that warrant consideration. First, it is important to acknowledge the potential for reverse causation in the observed relationship between CKM stage and frailty. While our results suggest that increased frailty is associated with more advanced stages of CKM, it is also plausible that frailty itself may arise as a consequence of physiological and functional decline associated with progressive kidney dysfunction. Due to the cross-sectional nature of our study, we cannot infer temporal directionality. Future longitudinal research is necessary to disentangle the causal pathways and determine whether frailty contributes to CKM progression, results from it, or both. Second, the calculation of the FI itself may have inherent limitations, including insufficient standardization of indicator selection and calculation methods. Additionally, some data collection may be complex, with missing key data or reliance on patient self-reports, potentially leading to bias or insufficient information. Furthermore, the static assessment of the FI may not fully capture the dynamic changes in a patient’s health status, possibly resulting in an imprecise evaluation of the true Frailty condition. Third, the study data were derived from the CKM population in NHANES, due to missing information and reliance on patient self-reports for some data, there may be misclassification of CKM stages, which could affect the accuracy of the study results. Fourth, although various potential confounding factors were adjusted for during the study, residual confounding factors may still exist. These unidentified or inadequately controlled confounders could affect the reliability of the study results. Fifth, the mortality outcomes assessed were limited to all-cause, cardiovascular, and non-cardiovascular death. Other clinically relevant endpoints such as hospitalisations, functional decline, or quality of life were not captured. Sixth, potentially relevant confounders, such as dietary factors, inflammation markers, and medication use, were not available in our dataset. Their absence may limit the completeness of our adjustment. Future studies with broader data coverage are needed to explore their role. Finally, the findings were derived from a U.S. population-based dataset. Thus, extrapolation to other populations or healthcare settings should be made with caution.

## 5. Conclusions

This study is the first to demonstrate a significant association between higher frailty burden, as measured by the FI, and both CKM stage progression and increased mortality risk. These findings highlight the frailty status as not merely a marker of vulnerability, but as a dynamic indicator of systemic dysfunction across cardiovascular, kidney, and metabolic domains. By capturing the cumulative health deficit, the frailty status offers prognostic value in identifying high-risk individuals and may serve as a stratification tool to tailor preventive and therapeutic strategies. Integrating frailty assessment into CKM management pathways could enhance early intervention, optimise resource allocation, and ultimately improve long-term clinical outcomes.

## Figures and Tables

**Table 1:** Baseline characteristics stratified by frailty status.

Characteristics	All (N = 19,089)	Robust (N = 3056)	Pre-Frail (N = 12,123)	Frail (N = 3910)	*p*
Age, years	63.00 (54.00, 72.00)	60.00 (51.00, 67.00)	63.00 (54.00, 72.00)	65.00 (56.00, 75.00)	<0.001
Male, n (%)	9741.00 (51.03%)	1858.00 (60.80%)	6161.00 (50.82%)	1722.00 (44.04%)	<0.001
Race and ethnicity, n (%)					<0.001
Non-Hispanic White	9552.00 (50.04%)	1564.00 (51.18%)	6094.00 (50.27%)	1894.00 (48.44%)	
Non-Hispanic Black	3841.00 (20.12%)	398.00 (13.02%)	2537.00 (20.93%)	906.00 (23.17%)	
Mexican American	2908.00 (15.23%)	545.00 (17.83%)	1792.00 (14.78%)	571.00 (14.60%)	
Hispanic and Others	2788.00 (14.61%)	549.00 (17.96%)	1700.00 (14.02%)	539.00 (13.79%)	
Body mass index, kg/m^2^	28.67 (25.36, 32.80)	27.25 (24.30, 30.84)	28.59 (25.40, 32.52)	30.10 (26.14, 35.40)	<0.001
Waist circumference, cm	101.40 (92.50, 111.10)	97.40 (89.20, 106.30)	101.20 (92.60, 110.50)	105.70 (96.30, 116.80)	<0.001
Poverty income ratio	2.29 (1.23, 4.31)	3.04 (1.63, 5.00)	2.52 (1.32, 4.52)	1.47 (0.92, 2.61)	<0.001
Education, n (%)					<0.001
Less than high school	2769.00 (14.51%)	392.00 (12.83%)	1612.00 (13.30%)	765.00 (19.57%)	
High school or equivalent	7337.00 (38.44%)	1025.00 (33.54%)	4590.00 (37.86%)	1722.00 (44.04%)	
College or above	8983.00 (47.06%)	1639.00 (53.63%)	5921.00 (48.84%)	1423.00 (36.39%)	
Marital status, n (%)					<0.001
Unmarried	1200.00 (6.29%)	157.00 (5.14%)	759.00 (6.26%)	284.00 (7.26%)	
Married	11,872.00 (62.19%)	2200.00 (71.99%)	7702.00 (63.53%)	1970.00 (50.38%)	
Divorcee	6017.00 (31.52%)	699.00 (22.87%)	3662.00 (30.21%)	1656.00 (42.35%)	
Smoking status, n (%)					<0.001
Never smoker	9261.00 (48.51%)	1648.00 (53.93%)	5991.00 (49.42%)	1622.00 (41.48%)	
Former smoker	6579.00 (34.46%)	891.00 (29.16%)	4256.00 (35.11%)	1432.00 (36.62%)	
Current smoker	3249.00 (17.02%)	517.00 (16.92%)	1876.00 (15.47%)	856.00 (21.89%)	
Alcohol consumption, n (%)					<0.001
Non-drinker	13,538.00 (70.92%)	1987.00 (65.02%)	8513.00 (70.22%)	3038.00 (77.70%)	
Mild to moderate	3777.00 (19.79%)	751.00 (24.57%)	2469.00 (20.37%)	557.00 (14.25%)	
Heavy	1774.00 (9.29%)	318.00 (10.41%)	1141.00 (9.41%)	315.00 (8.06%)	
Physical activity, n (%)					<0.001
Less than moderate	11,674.00 (61.16%)	1702.00 (55.69%)	7223.00 (59.58%)	2749.00 (70.31%)	
Moderate	4917.00 (25.76%)	798.00 (26.11%)	3278.00 (27.04%)	841.00 (21.51%)	
Vigorous	2498.00 (13.09%)	556.00 (18.19%)	1622.00 (13.38%)	320.00 (8.18%)	
Laboratory indicators					
Hemoglobin A1c, %	5.70 (5.40, 6.10)	5.50 (5.30, 5.70)	5.70 (5.40, 6.10)	6.00 (5.50, 6.80)	<0.001
Total Cholesterol, mg/dL	198.00 (171.00, 227.00)	209.00 (187.00, 236.00)	198.00 (171.00, 226.00)	186.50 (158.00, 219.00)	<0.001
HDL-C, mg/dL	50.00 (42.00, 62.00)	52.00 (43.00, 64.00)	51.00 (42.00, 63.00)	48.00 (40.00, 59.00)	<0.001
eGFR, ml/min/1.73 m^2^	82.69 (67.42, 95.94)	86.84 (74.35, 97.42)	83.17 (68.30, 96.05)	76.06 (57.20, 93.40)	<0.001
UACR, mg/g	8.67 (5.24, 19.74)	6.72 (4.42, 12.00)	8.54 (5.22, 18.45)	12.69 (6.48, 41.08)	<0.001
10-year CVD risk score	10.85 (5.13, 19.97)	6.49 (3.41, 11.80)	10.96 (5.25, 19.73)	15.89 (7.94, 26.45)	<0.001
CKM Stage					<0.001
CKM Stage 1	1215.00 (6.36%)	451.00 (14.76%)	711.00 (5.86%)	53.00 (1.36%)	
CKM Stage 2	11,294.00 (59.16%)	2320.00 (75.92%)	7488.00 (61.77%)	1486.00 (38.01%)	
CKM Stage 3	3151.00 (16.51%)	245.00 (8.02%)	2261.00 (18.65%)	645.00 (16.50%)	
CKM Stage 4	3429.00 (17.96%)	40.00 (1.31%)	1663.00 (13.72%)	1726.00 (44.14%)	

Abbreviations: CKM, cardiovascular-kidney-metabolic syndrome; CVD, cardiovascular disease; eGFR, estimated glomerular filtration rate; HDL-C, high-density lipoprotein cholesterol; UACR, urinary albumin to creatinine ratio. Robust: frailty index ≤ 0.08, Pre-frail: 0.08 < frailty index < 0.25, Frail: 0.25 ≤ frailty index.

**Table 2:** The interaction between Frailty Index and mortality outcomes in CKM patients.

	All-Cause Death		Cardiovascular Death		Non Cardiovascular Death	
	HR (95% CI)	*p*	HR (95% CI)	*p*	HR (95% CI)	*p*
			**Model I**			
Robust	*Reference*		*Reference*		*Reference*	
Pre-frail	1.92 (1.74, 2.13)	<0.001	2.37 (1.91, 2.94)	<0.001	1.80 (1.61, 2.02)	<0.001
Frail	4.10 (3.69, 4.56)	<0.001	5.92 (4.74, 7.40)	<0.001	3.61 (3.20, 4.08)	<0.001
			**Model II**			
Robust	*Reference*		*Reference*		*Reference*	
Pre-frail	1.47 (1.32, 1.62)	<0.001	1.71 (1.37, 2.12)	<0.001	1.40 (1.25, 1.57)	<0.001
Frail	2.83 (2.53, 3.17)	<0.001	3.78 (3.00, 4.76)	<0.001	2.57 (2.26, 2.91)	<0.001

Model I: Unadjusted; Model II: Adjusted age, sex, race and ethnicity, body mass index, waist circumference, poverty income ratio, marital states, education, smoking status, alcohol consumption, physical activity. Robust: frailty index ≤ 0.08, Pre-frail: 0.08 < frailty index < 0.25, Frail: 0.25 ≤ frailty index. Abbreviations: CI, confidence interval; CKM, cardiovascular-kidney-metabolic syndrome; HR, hazard ratio.

## Data Availability

The study leverages data sourced from the National Health and Nutrition Examination Survey (NHANES), a publicly accessible dataset (https://www.cdc.gov/nchs/nhanes/index.html [accessed on 23 August 2025]). The authors did not use any AI at all in the writing process.

## Supplementary Materials

The following supporting information can be downloaded at: https://www.mdpi.com/article/10.3390/jcm14176008/s1, Figure S1: Flowchart of this study; Figure S2: Distribution of mortality outcomes by CKM stages and frailty index in CKM patients; Figure S3: Subgroup analysis between frailty index and mortality outcomes; Table S1: Definitions of CKM; Table S2: Detailed algorithm for evaluating each CKM stage; Table S3: Detailed algorithm of the simplified 10-year cardiovascular disease risk models; Table S4: List of 49 variables included in the score of frailty index; Table S5: Baseline characteristics stratified by CKM stages; Table S6: Sensitivity analysis of different subgroups of FI and mortality outcomes in CKM patients; Table S7: Sensitivity analysis of Frailty Index and mortality outcomes in CKM patients.
