# Supplementary material for: Association of Frailty Status with Staging and Mortality Risk of Cardiovascular-Kidney-Metabolic Syndrome in Middle-Aged and Older Populations: Insights from the 1999–2018 National Health and Nutrition Examination Survey
Source: J Clin Med. 2025 Aug 25;14(17):6008. doi: 10.3390/jcm14176008 (PMC12429832; doi:10.3390/jcm14176008)
Supplement: Supplementary file 1 [file jcm-14-06008-s001.zip › jcm-3795211-supplementary.pdf]

Association of Frailty Status with Staging and Mortality Risk of Cardiovascular-Kidney-Metabolic Syndrome in Middle-aged and Older Populations: Insights from the 1999–2018 National Health and Nutrition Examination Survey

List of Supplementary Files

Supplementary Table S1. Definitions of CKM

Supplementary Table S2. Detailed algorithm for evaluating each CKM stage

Supplementary Table S3. Detailed algorithm of the simplified 10-year cardiovascular disease risk models

Supplementary Table S4. List of 49 variables included in the score of frailty index

Supplementary Table S5. Baseline characteristics stratified by CKM stages

Supplementary Table S6. Sensitivity analysis of different subgroups of FI and mortality outcomes in CKM patients

Supplementary Table S7. Sensitivity analysis of Frailty Index and mortality outcomes in CKM patients

Supplementary Figure S1. Flowchart of this study

Supplementary Figure S2. Distribution of mortality outcomes by CKM stages and frailty index in CKM patients

Supplementary Figure S3. Subgroup analysis between frailty index and mortality outcomes

Supplementary Table S1. Definitions of CKM

CKM conditions	Definition	CKM indicators	Threshold for CKM indicators
CVD	Individuals with clinical CVD or subclinical CVD	Clinical CVD	History of chronic heart failure, coronary heart disease, heart attack, or stroke
		Subclinical CVD	Any of the following criterion is met:<br>1) Very high-risk CKD in KDIGO classification: UACR ≥ 300 mg/g and eGFR ≤ 45-59 ml/min/1.73m2, UACR ≥ 30 mg/g and eGFR ≤ 30-44 ml/min/1.73m2, or eGFR ≤ 29 ml/min/1.73m2.<br>2) Predicted 10-year CVD risk ≥ 20%
Kidney diseases	Individuals with CKD	CKD	Moderate-to-high-risk CKD in KDIGO classification: UACR ≥ 30 mg/g and eGFR ≥ 60 ml/min/1.73m2, UACR < 300 mg/g and eGFR ≤ 45-59 ml/min/1.73m2, or UACR < 30 mg/g and eGFR ≤ 30-44 ml/min/1.73m2.
Metabolic disorders	Individuals with overweight/obesity, abdominal obesity, prediabetes, diabetes, hypertension, hypertriglyceridemia or MetS	Overweight/obesity	BMI ≥25 kg/m2 (or ≥23 kg/m2 if Asian ancestry) *
		Abdominal obesity	Waist circumference ≥88/102 cm in female/male (or if Asian ancestry ≥80/90 cm in female/male)
		Prediabetes	Fasting blood glucose ≥ 100-124 mg/dL or HbA1c ≥ 5.7%-6.4% and without self-reported diagnosis of diabetes, use of insulin, or oral hypoglycemic agents
		Diabetes	Fasting blood glucose ≥ 125 mg/dL or HbA1c ≥ 6.5% or self-reported diagnosis of diabetes, use of insulin, or oral hypoglycemic agents
		Hypertension	SBP ≥130 mm Hg or DBP ≥80 mm Hg or self-reported diagnosis of hypertension or use of antihypertensive medications
		Hypertriglyceridemia	Triglycerides ≥ 135 mg/dL
		MetS	MetS is defined by the presence of 3 or more of the following:<br>1) Waist circumference ≥88/102 cm in female/male (or if Asian ancestry ≥80/90 cm in female/male).<br>2) HDL cholesterol <50/40 mg/dL in female/male.

	3) Triglycerides ≥150 mg/dL.<br>4) Elevated blood pressure (SBP ≥130 mm Hg or DBP ≥80 mm Hg and/or use of antihypertensive medications)<br>5) Fasting blood glucose ≥100 mg/dL

*Asian was not listed as a separate race/ethnicity until NAHNES 2011-2012, therefore the uniform threshold for BMI and waist circumference was used in all participants in NHANES 1999-2010.

Abbreviations: BMI, body mass index; CKD, chronic kidney disease; CKM, cardiovascular-kidney-metabolic syndrom; CVD, cardiovascular disease; DBP, diastolic blood pressure; eGFR, estimated glomerular filtration rate; HDL-C, high-density lipoprotein cholesterol; KDIGO, The Kidney Disease: Improving Global Outcomes; MetS, metabolic syndrome; SBP, systolic blood pressure; UACR, urinary albumin to creatinine ratio.

Supplementary Table S2. Detailed algorithm for evaluating each CKM stage

**CKM stages**	**Definition**	**Criterion**	**Threshold for CKM conditions**
Stage 0: No CKM risk factors	Individuals with normal BMI and waist circumference, normoglycemia, normotension, a normal lipid profile, and no evidence of CKD or subclinical or clinical CVD	All criteria are met	BMI <25 kg/m^2 (or <23 kg/m^2 if Asian ancestry)*<br>Waist circumference <88/102 cm in female/male (or if Asian ancestry <80/90 cm in female/male)<br>Fasting blood glucose < 100 mg/dL and HbA1c < 5.7% and without self-reported diagnosis of diabetes, use of insulin, or oral hypoglycemic agents<br>SBP <130 mm Hg and DBP <80 mm Hg without self-reported diagnosis of hypertension or use of antihypertensive medications<br>HDL cholesterol >50/40 mg/dL in female/male and triglycerides < 150 mg/dL<br>Low-risk CKD in KDIGO classification according to eGFR and UACR: UACR < 30 mg/g and eGFR ≥ 60 ml/min/1.73m^2.<br>Predicted 10-year CVD risk < 20%<br>No clinical CVD
Stage 1: Excess or dysfunctional adiposity	Individuals with overweight/obesity, abdominal obesity, or dysfunctional adipose tissue, without the presence of other metabolic risk factors or CKD	Any of the three criteria is met	Overweight/obesity<br>Abdominal obesity<br>Prediabetes
		All criteria are met	SBP <130 mm Hg and DBP <80 mm Hg without self-reported diagnosis of hypertension or use of antihypertensive medications<br>HDL cholesterol >50/40 mg/dL in female/male and triglycerides <150 mg/dL<br>Low-risk CKD in KDIGO classification according to eGFR and UACR: UACR < 30 mg/g and eGFR ≥ 60 ml/min/1.73m^2<br>Predicted 10-year CVD risk < 20%<br>No clinical CVD
Stage 2: Metabolic risk factors and CKD	Individuals with metabolic risk factors (hypertriglyceridemia, hypertension, MetS, diabetes), or CKD	Any of the five criteria is met	Hypertriglyceridemia<br>Hypertension<br>diabetes<br>MetS<br>Moderate-to-high-risk CKD in KDIGO classification
		All criteria are met	No very high-risk CKD in KDIGO classification<br>Predicted 10-year CVD risk < 20%

			No clinical CVD
Stage 3: Subclinical CVD in CKM	Subclinical CVD among individuals with excess/dysfunctional adiposity, other metabolic risk factors, or CKD	Any of the two criteria is met	Very high-risk CKD in KDIGO classification<br>Predicted 10-year CVD risk ≥ 20%
		Any of the eight criteria is met	Overweight/obesity<br>Abdominal obesity<br>Prediabetes<br>Hypertriglyceridemia<br>Hypertension<br>diabetes<br>MetS<br>Moderate-to-high-risk CKD in KDIGO classification
		The criterion is met	No clinical CVD
Stage 4: Clinical CVD in CKM	Clinical CVD among individuals with excess/dysfunctional adiposity, other metabolic risk factors, or CKD	The criterion is met	Clinical CVD
		Any of the nine criteria is met	Overweight/obesity<br>Abdominal obesity<br>Prediabetes<br>Hypertriglyceridemia<br>Hypertension<br>diabetes<br>MetS<br>Moderate-to-high-risk CKD in KDIGO classification<br>Very high-risk CKD in KDIGO classification

*Asian was not listed as a separate race/ethnicity until NHANES 2011-2012, therefore the uniform threshold for BMI and waist circumference was used in all participants in NHANES 1999-2010.

Abbreviations: BMI, body mass index; CKD, chronic kidney disease; CKM, cardiovascular-kidney-metabolic syndrom; CVD, cardiovascular disease; DBP, diastolic blood pressure; eGFR, estimated glomerular filtration rate; HDL-C, high-density lipoprotein; KDIGO, The Kidney Disease: Improving Global Outcomes; NHANES, National Health and Nutrition Examination Survey; SBP, systolic blood pressure; UACR, urinary albumin to creatinine ratio.

Supplementary Table S3. Detailed algorithm of the simplified 10-year cardiovascular disease risk models

**Sex**	**Calculation**
Women	log-Odds = -3.307728 + 0.7939329 × (age – 55) /10 + 0.0305239 × (TC – HDL-C – 3.5) – 0.1606857 × (HDL-C – 1.3) /0.3 – 0.2394003 × (min(SBP, 110) – 110) /20 + 0.360078 × (max(SBP, 110) – 130) /20 + 0.8667604 × (if diabetes) + 0.5360739 × (if current smoker) + 0.6045917 × (min(eGFR, 60) – 60) / -15 + 0.0433769 × (max(eGFR, 60) – 90) / -15 + 0.3151672 × (if using anti-hypertensive medication) – 0.1477655 × (if using statin) – 0.0663612 × (if using anti-hypertensive medication) × (max(SBP, 110) – 130) /20 + 0.1197879 × (if using statin) × (TC – HDL-C – 3.5) – 0.0819715 × (age – 55) /10 × (TC – HDL-C – 3.5) + 0.0306769 × (age – 55) /10 × (HDL-C – 1.3) /0.3 – 0.0946348 × (age – 55) /10 × (max(SBP, 110) – 130) /20 – 0.27057 × (age – 55) /10 × (if diabetes) – 0.078715 × (age – 55) /10 × (if current smoker) – 0.1637806 × (age – 55) /10 × (min(eGFR, 60) – 60) / -15<br>Risk = exp(log-Odds) / (1 + exp(log-Odds))
Men	log-Odds = -3.031168 + 0.7688528 × (age – 55) /10 + 0.0736174 × (TC – HDL-C – 3.5) – 0.0954431 × (HDL-C – 1.3) /0.3 – 0.4347345 × (min(SBP, 110) – 110) /20 + 0.3362658 × (max(SBP, 110) – 130) /20 + 0.7692857 × (if diabetes) + 0.4386871 × (if current smoker) + 0.5378979 × (min(eGFR, 60) – 60) / -15 + 0.0164827 × (max(eGFR, 60) – 90) / -15 + 0.288879 × (if using anti-hypertensive medication) – 0.1337349 × (if using statin) – 0.0475924 × (if using anti-hypertensive medication) × (max(SBP, 110) – 130) /20 + 0.150273 × (if using statin) × (TC – HDL-C – 3.5) – 0.0517874 × (age – 55) /10 × (TC – HDL-C – 3.5) + 0.0191169 × (age – 55) /10 × (HDL-C – 1.3) /0.3 – 0.1049477 × (age – 55) /10 × (max(SBP, 110) – 130) /20 – 0.2251948 × (age – 55) /10 × (if diabetes) – 0.0895067 × (age – 55) /10 × (if current smoker) – 0.1543702 × (age – 55) /10 × (min(eGFR, 60) – 60) / -15<br>Risk = exp(log-Odds) / (1 + exp(log-Odds))

Abbreviations: eGFR, estimated glomerular filtration rate; HDL-C, high-density lipoprotein cholesterol; SBP, systolic blood pressure; TC, total cholesterol.

Supplementary Table S4. List of 49 variables included in the score of frailty index

Variable	Scoring	Proportion of missing value, n (%)
Cognition		
1. Experience confusion/memory problems	Yes = 1;<br>No = 0	10 (0.05%)
Dependence		
2. Managing money	No difficulty = 0;<br>Some difficulty = 0.33;<br>Much difficulty = 0.66;<br>Unable to do = 1	11,287 (52.23%)
3. Stooping, crouching, kneeling difficulty	No difficulty = 0;<br>Some difficulty = 0.33;<br>Much difficulty = 0.66;<br>Unable to do = 1	11,062 (51.19%)
4. Lifting or carrying difficulty	No difficulty = 0;<br>Some difficulty = 0.33;<br>Much difficulty = 0.66;<br>Unable to do = 1	11,079 (51.27%)
5. House chore difficulty	No difficulty = 0;<br>Some difficulty = 0.33;<br>Much difficulty = 0.66;<br>Unable to do = 1	11,209 (51.87%)
6. Preparing meals difficulty	No difficulty = 0;<br>Some difficulty = 0.33;<br>Much difficulty = 0.66;<br>Unable to do = 1	11,289 (52.24%)
7. Standing up from armless chair difficulty	No difficulty = 0;<br>Some difficulty = 0.33;<br>Much difficulty = 0.66;<br>Unable to do = 1	10,989 (50.85%)
8. Getting in and out of bed difficulty	No difficulty = 0;<br>Some difficulty = 0.33;<br>Much difficulty = 0.66;<br>Unable to do = 1	10,993 (50.87%)
9. Using fork, knife, drinking from cup difficulty	No difficulty = 0;<br>Some difficulty = 0.33;	10,986 (50.84%)

	Much difficulty = 0.66;<br>Unable to do = 1	
10. Dressing yourself difficulty	No difficulty = 0;<br>Some difficulty = 0.33;<br>Much difficulty = 0.66;<br>Unable to do = 1	10,990 (50.86%)
11. Standing for long periods difficulty	No difficulty = 0;<br>Some difficulty = 0.33;<br>Much difficulty = 0.66;<br>Unable to do = 1	11,131 (51.51%)
12. Grasp/holding small objects difficulty	No difficulty = 0;<br>Some difficulty = 0.33;<br>Much difficulty = 0.66;<br>Unable to do = 1	10,994 (50.88%)
13. Attending social event difficulty	No difficulty = 0;<br>Some difficulty = 0.33;<br>Much difficulty = 0.66;<br>Unable to do = 1	11,307 (52.33%)
14. Push or pull large objects difficulty	No difficulty = 0;<br>Some difficulty = 0.33;<br>Much difficulty = 0.66;<br>Unable to do = 1	13,082 (60.54%)
15. Walking for a quarter mile difficulty	No difficulty = 0;<br>Some difficulty = 0.33;<br>Much difficulty = 0.66;<br>Unable to do = 1	12,435 (57.55%)
16. Walking up 10 steps difficulty	No difficulty = 0;<br>Some difficulty = 0.33;<br>Much difficulty = 0.66;<br>Unable to do = 1	12,411 (57.43%)
17. Leisure activity at home difficulty	No difficulty = 0;<br>Some difficulty = 0.33;<br>Much difficulty = 0.66;<br>Unable to do = 1	11,001 (50.91%)
Depressive Symptoms		

18. Have little interest in doing things	1999-2004: Every day, nearly every day = 1, Most days = 0.75, about half the days = 0.50, less than half the days = 0.25, Not at all =0; 2005-2018: Nearly every day = 1, More than half the days = 0.66, Several days = 0.33, Not at all = 0	5,751 (26.61%)
19. Feeling down, depressed, or hopeless	1999-2004: Every day, nearly every day = 1, Most days = 0.75, about half the days = 0.50, less than half the days = 0.25, Not at all =0; 2005-2018: Nearly every day = 1, More than half the days = 0.66, Several days = 0.33, Not at all = 0	5,671 (26.24%)
20. Trouble sleeping or sleeping too much	1999-2004: Every night = 1, Nearly every night = 0.66, less often = 0.33, Not at all = 0 2005-2018: Nearly every day = 1, More than half the days = 0.66, Several days = 0.33, Not at all = 0	6,308 (29.19%)
21. Feeling tired or having little energy	Nearly every day = 1, More than half the days = 0.66, Several days = 0.33, Not at all = 0	6,413 (29.68%)
22. Poor appetite or overeating	1999-2004: Yes = 1, No = 0 2005:2018: Nearly every day = 1, More than half the days = 0.66, Several days = 0.33, Not at all = 0	6,305 (29.18%)
23. Feeling bad about yourself	1999-2004: Yes = 1, No = 0 2005:2018: Nearly every day = 1, More than half the days = 0.66, Several days = 0.33, Not at all = 0	6,312 (29.21%)
24.Trouble concentrating on things	1999-2004: Yes = 1, No = 0 2005:2018: Nearly every day = 1, More than half the days = 0.66, Several days = 0.33, Not at all = 0	6,309 (29.20%)
Comorbidities		
25. Arthritis	Yes = 1, No = 0	32 (0.15%)
26. Thyroid problems	Yes = 1, No = 0	33 (0.15%)
27. Chronic bronchitis	Yes = 1, No = 0	43 (0.20%)
28. Cancer	Yes = 1, No = 0	11 (0.05%)
29. Congestive heart failure	Yes = 1, No = 0	34 (0.16%)
30. Coronary heart disease	Yes = 1, No = 0	43 (0.20%)
31. Angina/Angina pectoris	Yes = 1, No = 0	58 (0.27%)
32. Heart attack	Yes = 1, No = 0	18 (0.08%)
33. Stroke	Yes = 1, No = 0	3 (0.01%)
34. Hypertension	Yes = 1, No = 0	58 (0.27%)
35. Diabetes	Yes = 1, No = 0	4 (0.02%)

36. Weak/failing kidneys	Yes = 1, No = 0	29 (0.13%)
37. Urinary Leakage	1999-2000: Yes = 1, No = 0<br>2001-2018: Greatly = 1, Very much = 0.75, Somewhat = 0.5, Only a little = 0.25	13,847 (64.08%)
Hospital Utilization and Access to Care		
38. Self-rated health	Fair, poor = 1, Excellent, Very good, good = 0	No missing value
39. Health now compared with 1 year ago	Worse = 1, About the same, better = 0	No missing value
40. Overnight hospital patient in past year	Yes = 1, No = 0	8 (0.04%)
41. Frequency of health care use during past year	None = 0, 1-4 = 0.5, 5 and more = 1	9 (0.04%)
42. Number of prescribed medications	None = 0, 1-4 = 0.5, 5 and more = 1	No missing value
Physical Anthropometry		
43. Body mass index	< 18.5, ≥30 = 1<br>25-30 = 0.5<br>18.5-25 = 0	No missing value
Laboratory Values		
44. Glycohemoglobin (%)	0%-5.7% = 0, >5.7% = 1	36 (0.17%)
45. Red blood cell count (million cells/mL)	MALE: 4.7-6.1 = 0, Other = 1<br>FEMALE: 4.2-5.4 = 0, Other = 1	41 (0.19%)
46. Hemoglobin (g/dL)	MALE: 13.5-18 = 0, Other = 1<br>FEMALE: 12-16 = 0, Other = 1	41 (0.19%)
47. Red cell distribution width (%)	11.6-14.6 = 0, Other = 1	41 (0.19%)
48. Lymphocyte percent (%)	20-40 = 0, Other = 1	80 (0.37%)
49. Segmented neutrophils percent (%)	40-80 = 0, Other = 1	80 (0.37%)

For participants with missing data on certain frailty-related items, the frailty score was calculated by dividing the total score of available frailty-related items by the total number of items for which the participant had data.

Supplementary Table S5. Baseline characteristics stratified by CKM stages

**Characteristics**	**All (n = 19,407)**	**Non-CKM (n = 318)**	**CKM Stage 1 (n = 1,215)**	**CKM Stage 2 (n = 11,294)**	**CKM Stage 3 (n = 3,151)**	**CKM Stage 4 (n = 3,429)**	***P***
Age, years	63.00 (54.00, 72.00)	52.00 (48.00, 59.00)	54.00 (49.00, 62.00)	59.00 (52.00, 65.00)	78.00 (72.00, 80.00)	70.00 (62.00, 78.00)	< 0.001
Male, n (%)	9,853.00 (50.77%)	112.00 (35.22%)	576.00 (47.41%)	5,260.00 (46.57%)	1,818.00 (57.70%)	2,087.00 (60.90%)	< 0.001
Race, n (%)							< 0.001
Non-Hispanic White	9,772.00 (50.35%)	220.00 (69.18%)	583.00 (47.98%)	5,008.00 (44.34%)	1,914.00 (60.74%)	2,047.00 (59.70%)	
Non-Hispanic Black	3,873.00 (19.96%)	32.00 (10.06%)	189.00 (15.56%)	2,468.00 (21.85%)	525.00 (16.66%))	659.00 (19.22%)	
Mexican American	2,935.00 (15.12%)	27.00 (8.49%)	198.00 (16.30%)	1,944.00 (17.21%)	407.00 (12.92%)	359.00 (10.47%)	
Hispanic and Others	2,827.00 (14.57%)	39.00 (12.26%)	245.00 (20.16%)	1,874.00 (16.59%)	305.00 (9.68%)	364.00 (10.62%)	
Body mass index, kg/m^2	28.52 (25.16, 32.70)	22.23 (20.51, 23.61)	27.11 (24.84, 30.20)	29.00 (25.56, 33.30)	27.93 (24.79, 31.50)	29.00 (25.60, 33.30)	< 0.001
Waist circumference, cm	101.20 (92.10, 111.00)	80.90 (76.00, 85.50)	95.80 (88.50, 103.90)	101.10 (92.10, 111.00)	102.20 (93.50, 110.80)	104.10 (95.50, 114.30)	< 0.001
Poverty income ratio	2.31 (1.24, 4.35)	4.18 (1.83, 5.00)	3.16 (1.64, 5.00)	2.56 (1.27, 4.69)	1.96 (1.20, 3.35)	1.84 (1.11, 3.41)	< 0.001
Education, n (%)							< 0.001
Less than high school	2,787.00 (14.36%)	18.00 (5.66%)	129.00 (10.62%)	1,440.00 (12.75%)	646.00 (20.50%)	554.00 (16.16%)	
High school or equivalent	7,414.00 (38.20%)	77.00 (24.21%)	351.00 (28.89%)	4,254.00 (37.67%)	1,255.00 (39.83%)	1,477.00 (43.07%)	
College or above	9,206.00 (47.44%)	223.00 (70.13%)	735.00 (60.49%)	5,600.00 (49.58%)	1,250.00 (39.67%)	1,398.00 (40.77%)	
Marital status, n (%)							< 0.001
Unmarried	1,226.00 (6.32%)	26.00 (8.18%)	87.00 (7.16%)	831.00 (7.36%)	109.00 (3.46%)	173.00 (5.05%)	
Married	12,092.00 (62.31%)	220.00 (69.18%)	847.00 (69.71%)	7,263.00 (64.31%)	1,763.00 (55.95%)	1,999.00 (58.30%)	
Divorcee	6,089.00 (31.38%)	72.00 (22.64%)	281.00 (23.13%)	3,200.00 (28.33%)	1,279.00 (40.59%)	1,257.00 (36.66%)	
Smoking status, n (%)							< 0.001
Never smoker	9,434.00 (48.61%)	173.00 (54.40%)	642.00 (52.84%)	5,769.00 (51.08%)	1,550.00 (49.19%)	1,300.00 (37.91%)	
Former smoker	6,651.00 (34.27%)	72.00 (22.64%)	372.00 (30.62%)	3,337.00 (29.55%)	1,378.00 (43.73%)	1,492.00 (43.51%)	
Current smoker	3,322.00 (17.12%)	73.00 (22.96%)	201.00 (16.54%)	2,188.00 (19.37%)	223.00 (7.08%)	637.00 (18.58%)	
Alcohol consumption, n (%)							< 0.001

Non-drinker	13,731.00 (70.75%)	193.00 (60.69%)	772.00 (63.54%)	7,363.00 (65.19%)	2,688.00 (85.31%)	2,715.00 (79.18%)	
Mild to moderate	3,868.00 (19.93%)	91.00 (28.62%)	318.00 (26.17%)	2,620.00 (23.20%)	356.00 (11.30%)	483.00 (14.09%)	
Heavy	1,808.00 (9.32%)	34.00 (10.69%)	125.00 (10.29%)	1,311.00 (11.61%)	107.00 (3.40%)	231.00 (6.74%)	
Physical activity, n (%)							< 0.001
Less than moderate	11,850.00 (61.06%)	176.00 (55.35%)	694.00 (57.12%)	6,723.00 (59.53%)	2,053.00 (65.15%)	2,204.00 (64.28%)	
Moderate	4,997.00 (25.75%)	80.00 (25.16%)	305.00 (25.10%)	2,870.00 (25.41%)	848.00 (26.91%)	894.00 (26.07%)	
Vigorous	2,560.00 (13.19%)	62.00 (19.50%)	216.00 (17.78%)	1,701.00 (15.06%)	250.00 (7.93%)	331.00 (9.65%)	
Laboratory indicators							
Hemoglobin A1c, %	5.70 (5.40, 6.10)	5.30 (5.10, 5.40)	5.50 (5.30, 5.80)	5.70 (5.40, 6.00)	5.80 (5.50, 6.60)	5.80 (5.50, 6.50)	< 0.001
Total Cholesterol, mg/dL	198.00 (171.00, 227.00)	204.00 (181.00, 227.00)	203.00 (182.00, 223.00)	205.00 (178.00, 233.00)	190.00 (164.00, 217.00)	178.00 (152.00, 210.00)	< 0.001
HDL-C, mg/dL	51.00 (42.00, 62.00)	67.00 (57.00, 78.00)	60.00 (52.00, 70.00)	51.00 (42.00, 63.00)	49.00 (41.00, 60.00)	47.00 (39.00, 58.00)	< 0.001
eGFR, ml/min/1.73m^2	82.89 (67.66, 96.08)	91.91 (81.03, 101.53)	92.22 (82.16, 102.07)	88.75 (75.47, 99.46)	64.86 (50.60, 79.32)	71.14 (54.82, 86.36)	< 0.001
UACR, mg/g	8.61 (5.21, 19.39)	5.91 (4.25, 9.14)	5.50 (3.93, 8.22)	7.75 (4.97, 15.27)	14.30 (7.24, 39.39)	11.88 (6.34, 36.54)	< 0.001
10-year CVD risk score	10.65 (4.97, 19.79)	2.71 (1.63, 4.71)	3.60 (2.19, 6.47)	7.64 (4.14, 12.53)	26.05 (22.78, 30.68)	19.40 (11.01, 28.01)	< 0.001
Frailty Index	0.15 (0.10, 0.23)	0.09 (0.06, 0.12)	0.10 (0.07, 0.14)	0.14 (0.09, 0.20)	0.17 (0.12, 0.23)	0.25 (0.18, 0.34)	< 0.001

Abbreviations: CKM, cardiovascular-kidney-metabolic syndrome; CVD, cardiovascular disease; eGFR, estimated glomerular filtration rate; HDL-C, high-density lipoprotein cholesterol; UACR, urinary albumin to creatinine ratio.

Supplementary Table S6. Sensitivity analysis of different subgroups of FI and mortality outcomes in CKM patients

	All-cause mortality		Cardiovascular mortality		Non-cardiovascular mortality	
	**HR (95% CI)**	***P***	**HR (95% CI)**	***P***	**HR (95% CI)**	***P***
FI ≤0.10	*Reference*		*Reference*		*Reference*	
0.10< FI <0.25	1.52 (1.40, 1.66)	< 0.001	1.73 (1.45, 2.06)	< 0.001	1.46 (1.33, 1.61)	< 0.001
FI ≥0.25	2.82 (2.57, 3.10)	< 0.001	3.65 (3.02, 4.40)	< 0.001	2.57 (2.31, 2.87)	< 0.001
FI ≤0.10	*Reference*		*Reference*		*Reference*	
0.10< FI <0.21	1.42 (1.30, 1.55)	< 0.001	1.57 (1.31, 1.88)	< 0.001	1.38 (1.25, 1.52)	< 0.001
FI ≥0.21	2.53 (2.32, 2.77)	< 0.001	3.23 (2.69, 3.87)	< 0.001	2.33 (2.10, 2.58)	< 0.001

Models were adjusted for age, sex, race and ethnicity, body mass index, waist circumference, poverty income ratio, marital states, education, smoking status, alcohol consumption, physical activity.

Abbreviations: CI, confidence interval; CKM, cardiovascular-kidney-metabolic syndrome; FI, frailty index; HR, hazard ratio.

Supplementary Table S7. Sensitivity analysis of Frailty Index and mortality outcomes in CKM patients

	All-cause mortality		Cardiovascular mortality		Non-cardiovascular mortality	
	**HR (95% CI)**	***P***	**HR (95% CI)**	***P***	**HR (95% CI)**	***P***
**Without history of cancer**						
Robust	*Reference*		*Reference*		*Reference*	
Pre-frail	1.47 (1.32, 1.63)	< 0.001	1.79 (1.42, 2.24)	< 0.001	1.38 (1.22, 1.56)	< 0.001
Frail	2.76 (2.45, 3.11)	< 0.001	4.03 (3.16, 5.15)	< 0.001	2.40 (2.09, 2.76)	< 0.001
**Died within the first two-year follow-up**						
Robust	*Reference*		*Reference*		*Reference*	
Pre-frail	1.44 (1.29, 1.60)	< 0.001	1.65 (1.32, 2.06)	< 0.001	1.38 (1.22, 1.55)	< 0.001
Frail	2.67 (2.38, 3.00)	< 0.001	3.48 (2.73, 4.42)	< 0.001	2.44 (2.14, 2.79)	< 0.001

Models were adjusted for age, sex, race and ethnicity, body mass index, waist circumference, poverty income ratio, marital states, education, smoking status, alcohol consumption, physical activity.

Robust: frailty index ≤0.08; Pre-frail: 0.08< frailty index <0.25; Frail: 0.25≤ frailty index.

Abbreviations: CI, confidence interval; CKM, cardiovascular-kidney-metabolic syndrome; HR, hazard ratio.

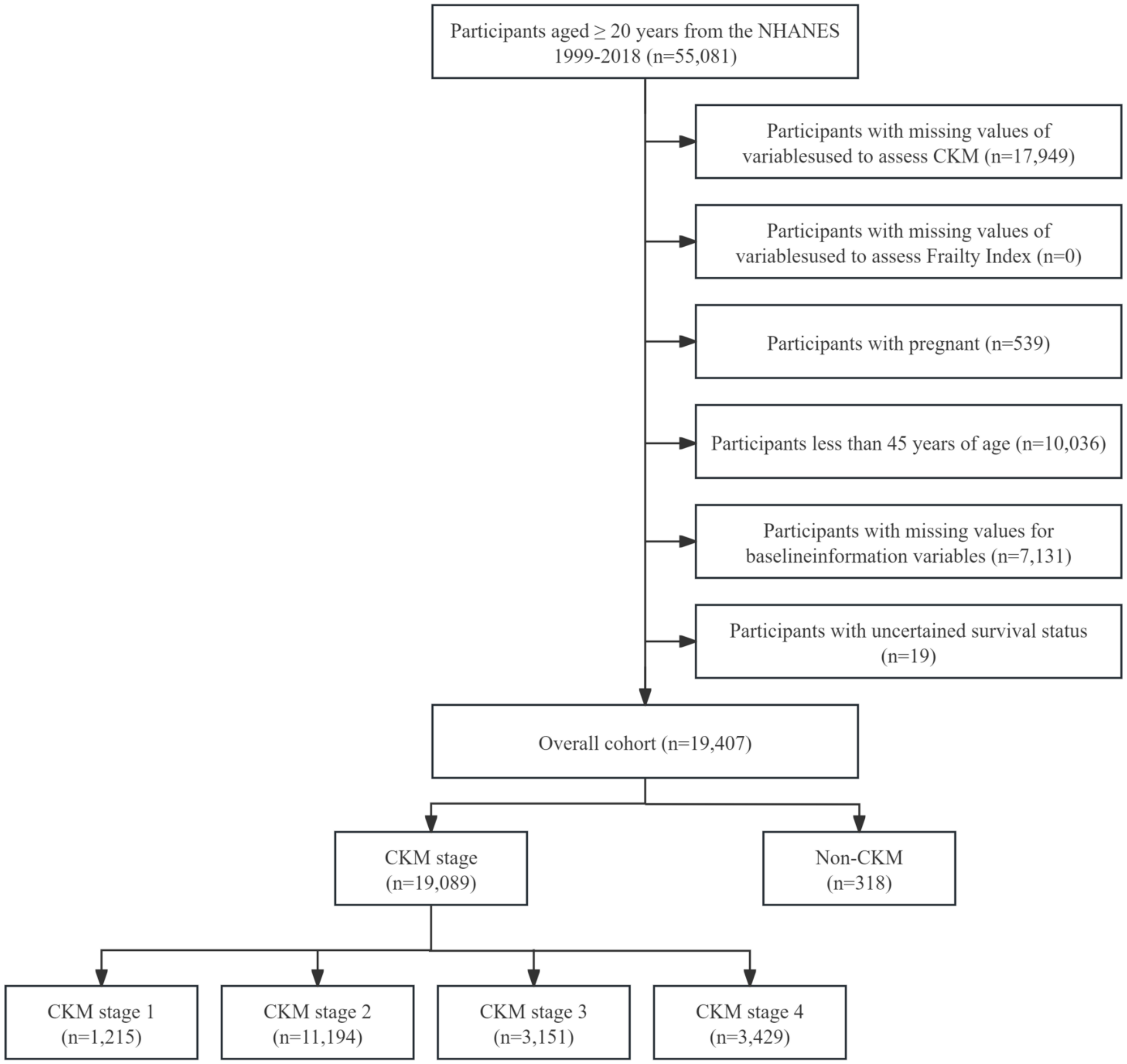

Supplementary Figure S1. Flowchart of this study. CKM, cardiovascular-kidney-metabolic syndrome; NHANES, National Health and Nutrition Examination Survey.

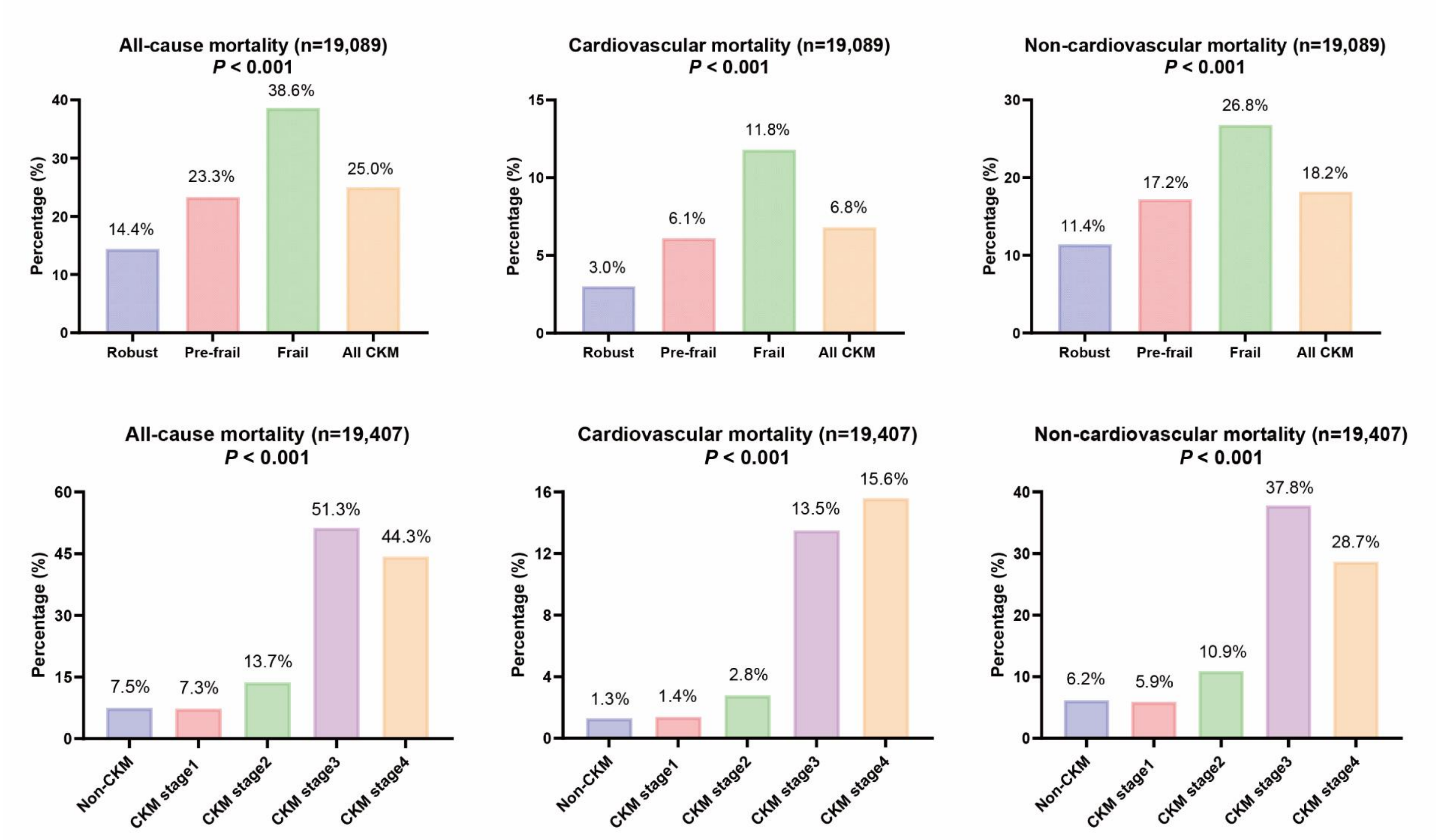

Supplementary Figure S2. Distribution of mortality outcomes by CKM stages and frailty index in CKM patients. P values from the Fisher's exact test. CKM, cardiovascular-kidney-metabolic syndrome.

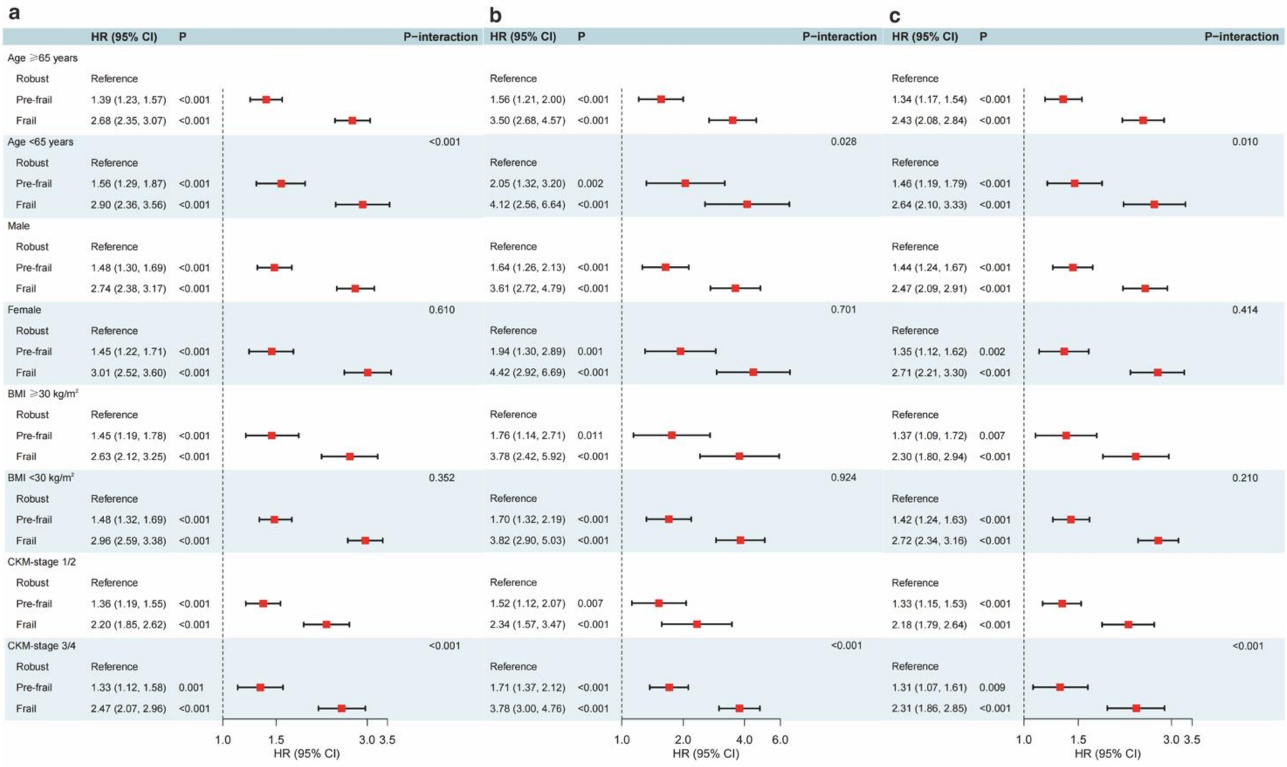

Supplementary Figure S3. Subgroup analysis between frailty index and mortality outcomes. (a) All-cause mortality, (b) cardiovascular mortality, (c) non-cardiovascular mortality from NHANES. P values from multivariable Cox proportional hazards models. CI, confidence interval; CKM, cardiovascular-kidney-metabolic syndrome; HR, hazard ratio.
